# Neurointerventions for Criminal Offenders: Psychological Connectedness, Culpability and Justified Punishment

**DOI:** 10.1007/s10677-025-10526-8

**Published:** 2025-11-04

**Authors:** Vera Tesink

**Affiliations:** Department of Philosophy, Faculty of Humanities, Vrije Universiteit Amsterdam, Amsterdam, Netherlands

**Keywords:** Neurointerventions, Personal identity, Psychological connectedness, Culpability, Punishment

## Abstract

Neurointerventions may be employed in criminal justice as rehabilitative tools that aim to reduce reoffending. Although ethical debates have concentrated largely on the effects of these interventions on autonomy, bodily integrity and mental integrity, much less attention has been paid to their potential impact on personal identity. On a Parfitian view of identity as psychological connectedness, neurointerventions, by modifying offenders’ psychological traits and dispositions, risk weakening offenders’ psychological connections to their earlier selves. By reducing psychological connections—and by acting directly on the very psychological traits and dispositions implicated in past crimes—neurointerventions could significantly diminish culpability that is grounded in those connections. As most penal systems set the scope of justified punishment in proportion to an offender’s culpability, any neurointervention-induced reduction in psychological connectedness may render further punishment unjust insofar as it becomes disproportionate to current culpability. This suggests that post-neurointervention reassessment of sentences may be warranted to keep punishment proportional to current culpability—and thus morally justified.

## Introduction

Advancements in neurotechnology over the past decades have greatly expanded our ability to modify brain function. Technologies such as transcranial direct current stimulation (tDCS) and deep brain stimulation (DBS) can modulate neural signaling and thereby alter a person’s mental states and behavior. In recent years, there has been increasing interest in the potential use of such neurotechnologies within the criminal justice system as rehabilitative tools to change offenders’ mental states and behavior and reduce the likelihood of reoffending (Ryberg 2020). Within criminal justice, so-called ‘neurointerventions’ are defined as “procedures that produce a physical, chemical, or biological effect on the brain to reduce the likelihood … of criminal offending” (Birks & Douglas 2018). Some neurointerventions are already employed in Europe and the United States, such as anti-libidinal interventions (or ‘chemical castration’) that use psychoactive drugs to suppress testosterone in adult men to pre-adolescent levels to decrease the risk of recidivism among sex offenders (Forsberg 2021b). Developments in neuroscience may increase the number of available technologies that directly target the brain and could reduce recidivism among a wider range of offenders. For instance, aggression reduction via tDCS has already been reported in forensic settings (Hofhansel et al. 2020; Sergiou et al. 2020).

The use of neurointerventions in criminal justice settings raises ethical concerns. Concerns discussed in the literature primarily pertain to autonomy (Douglas et al. 2013; Ligthart et al. 2021), bodily integrity (Douglas 2014; Tesink et al. 2023) and mental integrity (Craig 2016; Shaw 2018). While receiving some scholarly attention (Holmen 2022), less extensively discussed are potential effects of neurointerventions on offenders’ personal identity. On Parfit’s influential account of personal identity (1984), identity over time is treated as overlapping psychological connections—such as beliefs, desires and intentions—one bears to future or past selves. Understood this way, it is clear that neurointerventions—by changing psychological states and traits—might affect identity by weakening psychological connections over time. According to Parfit, psychological connectedness underlies important moral concepts and practices such as acting in one’s own future interest and responsibility for past actions, as we have reason to care about our future wellbeing or to accept blame for past deeds only insofar as overlapping memories, intentions and values persist across time that make the later or earlier person meaningfully ‘us’ (Parfit 1984).

Especially the latter is an important consideration within criminal justice contexts, as responsibility grounds moral culpability, or blameworthiness for past wrongdoing, which in turn is often considered important for determining the limits of justified punishment. That is, most penal theories accept that there is a culpability-based upper limit to justified punishment, where the extent of punishment that can justifiably be imposed should be proportionate to the extent of culpability for a crime (Duff 2000; Walen 2021). Upon reducing psychological connections in an offender by changing psychological states and traits, neurointerventions might reduce culpability and thereby also the extent of punishment that is morally justifiable.

Accordingly, this article argues that if neurointerventions reduce psychological connectedness and culpability in criminal offenders, any further punishment imposed post-neurointervention might become disproportionate to culpability and therefore less morally justifiable. The next section discusses neurointervention use in criminal justice and their potential effects on identity. The third section introduces Parfit’s account of personal identity understood as psychological connectedness and explains how it underlies moral culpability. The fourth section considers how neurointerventions might diminish culpability in criminal offenders by weakening psychological connections and modifying exactly those connections that ground culpability. The fifth section explains how culpability is relevant to setting justified punishments. The sixth and final section argues that neurointervention-induced weakening of psychological connectedness and culpability may render further punishment morally unjustified insofar as it is disproportionate to the offender’s current culpability, and considers some objections.

## Neurointerventions and Personal Identity

Neurointerventions may be employed in criminal justice contexts to reduce recidivism risk.^1^ They could be used to target psychological states and traits that are associated with previous criminal behavior, and by modifying these, reduce the likelihood that an offender will commit another offense. In most proposals, such interventions are presented as rehabilitative measures that have the potential to ‘reform’ offenders by addressing traits and dispositions—such as impulse control problems—that contributed to prior offending (Pugh and Douglas 2016). If implemented, neurointerventions would likely be offered in addition to a prison sentence, in exchange for a reduced sentence or as a condition of early release.^2^

The use of neurointerventions in criminal justice raises ethical concerns. In the literature, concerns are addressed regarding infringements of offenders’ rights, such as the right to autonomy (Douglas et al. 2013; Ligthart et al. 2021), bodily integrity (Douglas 2014; Tesink et al. 2023) and mental integrity (Craig 2016; Shaw 2018). Less often discussed within the context of criminal justice are the effects that neurointerventions might have on personal identity. In the medical literature, studies have reported significant changes to psychological states and traits, often as a byproduct of neurotechnology use for the treatment of neurological disorders such as Parkinson’s disease (PD). For instance, it was reported that a number of PD patients who underwent DBS experienced changes such as increased apathy or hypersexuality (Schüpbach et al. 2006), which the researchers claim could threaten patients’ autonomy, authenticity and agency (Schüpbach et al. 2006). Another study of PD patients who received DBS observed increased impulsivity and self-centeredness, as well as decreased persistence and consciousness, three months after the DBS treatment started (Lipsman et al. 2009). Thomson and colleagues (2020) found that patients reported both positive and negative personality changes following DBS, with some attributing these changes to the stimulation, and the authors claim that these effects can impact patients’ relationships and self-perception and potentially lead to psychological distress or social difficulties (Thomson et al. 2020). In a similar vein, non-invasive neuromodulation such as tDCS has also been shown to affect potentially identity-relevant dispositions, for example by enhancing altruistic behavior or by changing moral evaluations of certain acts (Choy et al. 2018; Zheng et al. 2016). Within the neuroethical literature, scholars such as Baylis (2013), Klaming and Haselager (2013), Lipsman and Glannon (2013), Galert (2015) and Pugh (2020) have examined the moral implications of ‘identity changes’ after neurotechnology use, with most concluding that—despite differences in focus on numerical versus narrative identity—serious ethical concerns can arise, for example regarding autonomy or moral and prudential relationships.^3^ Given that similar neurotechnologies might serve as neurointerventions within criminal justice contexts, comparable effects on personal identity might be expected to occur here.

## Personal Identity as Psychological Connectedness

Before proceeding, it should be clarified what is understood as ‘personal identity’ and why it is morally relevant. According to the influential account of psychological continuity advanced by Parfit (1984) and relied on here, personal identity over time is treated as a matter of overlapping psychological connections, such as memories, intentions and beliefs. Such connections might for instance consist in remembering something at time T^2^ that happened at time T^1^, or forming an intention at time T^1^ and executing this intention at time T^2^. On this view, a future person is ‘you’ if there is a continuous chain of connected psychological states linking that future individual back to your current self. Importantly, for Parfit, no single thread of strict numerical identity is itself of fundamental moral importance. Instead, all that ‘matters’ in survival is that there is sufficient overlap in psychological connections across time, and as long as your future self retains enough of these connections, there is psychological continuity and you ‘survive’ (Parfit 1984).

According to Parfit, the moral and prudential reasons we typically associate with identity—such as responsibility for one’s past actions and caring for one’s own future welfare—are best explained by the extent of psychological connectedness (Parfit 1984). When psychological connections weaken significantly, as might happen with for instance severe amnesia, the usual rationales for treating that future individual as ‘oneself’ begin to lose force. Instead, the moral relationship that exists between the individual at one time and the—weakly psychologically connected—individual at another time might more closely resemble the relationship between two different people.

Importantly, Parfit’s emphasis on *degrees* of psychological connectedness opposes the traditional understanding of identity as an all-or-nothing phenomenon. In the case that more than half of someone’s psychological connections were to be severed, we might say they effectively become ‘someone else’ in a morally significant sense.^4^ However, a less drastic reduction in psychological connections need not entail that a completely new person is created. Rather, it results in a weaker form of connectedness or, as Parfit sometimes describes it, ‘partial survival’ (Parfit 1984). When the overlap in psychological connections is only partially diminished, the individual remains partially the same person, but the strength of the identity link—the degree to which it makes sense to say the person has completely ‘survived’—is also diminished. This is important for concepts such as moral responsibility, since in cases where an individual only partially ‘survives’ changes in core psychological traits, it might affect how strongly we should hold them responsible for past actions (without completely eliminating responsibility).^5^

If psychological connectedness underlies moral responsibility, it plausibly also underlies moral culpability (Douglas 2019). Culpability refers to being held responsible for a wrongful act or omission in a way that justifies moral condemnation—in other words, blameworthiness for wrongdoing. As psychological connections between a person at time T^1^ and time T^2^ weaken, the culpability of the person at T^2^ for wrongdoing committed at T^1^ may diminish. Parfit indeed claims that the basis for holding someone responsible may erode if the overlap of relevant psychological connections becomes too weak (Parfit 1984). We might think that the extent of culpability reduction would track the extent of psychological disconnection, where modest psychological disconnection between a person at T^1^ and T^2^ would result in diminished culpability, and complete psychological disconnection would eliminate culpability entirely. Alternatively, however, some may contend that there might be relevant thresholds, where for instance culpability only starts to diminish once psychological connectedness falls beneath a specific threshold, or culpability could remain absent even if there is some (minimal) threshold level of psychological connectedness. In any case, on the account of psychological connectedness as generally understood, it seems plausible that any marked reduction of the psychological connections between the person who acted and the person who now exists would lead to a reduction in culpability.^6^

## How Neurointerventions Can Weaken Psychological Connectedness—and Culpability

By altering an offender’s psychological traits and dispositions, neurointerventions could reduce psychological connectedness between an offender pre-intervention and post-intervention. Neurointerventions work by modulating brain activity, and when used on criminal offenders, they will likely target brain regions associated with previous criminal behavior. Think of brain regions involved in impulse control, emotion regulation or aggression. For example, tDCS might be used to reduce impulsive behavior by enhancing the functioning of a hypoactive prefrontal cortex, which has been linked to poor impulse control in certain offenders (Hofhansel et al. 2020), or DBS could be used to target specific brain structures involved in emotion regulation and decision-making to diminish aggressive tendencies (Escobar Vidarte et al. 2022; Harat et al. 2021).^7^ Depending on how central the targeted psychological traits and dispositions associated with previous criminal behavior are to the offender’s identity, neurointerventions can, to varying degrees, reduce the psychological connections the offender after the neurointervention bears to the offender before the neurointervention. If psychological connections are weakened between the ‘crime-committing self’ and the ‘post-neurointervention self,’ this could also imply a weakening of the continuity of the offender’s blameworthiness for the crime—and thus diminished culpability.^8^

One could argue that if the psychological traits and dispositions that the neurointervention changes are more ‘peripheral’ traits and dispositions that are not strongly associated with the offender’s identity—such as transient moods or episodic preferences that the offender does not endorse consistently—then much of the ‘important’ psychological connections remain intact and the effects on culpability would therefore likely be modest. However, when used on criminal offenders to modify or eliminate certain psychological states and behaviors that are strongly associated with past criminal acts to prevent reoffending, neurointerventions would plausibly target *exactly* those traits and dispositions that are relevant to culpability.

That is, culpability is generally tied to the psychological features that underlie a person’s wrongful actions (Moore 1996). This refers not only to the psychological or behavioral dispositions that directly resulted in a certain criminal act, such as heightened aggression, but also to psychological faculties such as impulse control, empathy, moral reasoning and recognition of the harm associated with certain actions (Segev 2025). This is because blameworthiness is often defined in terms of an epistemic condition—did the person understand the facts and moral stakes?—and a control condition—could they have regulated their conduct accordingly?—and meeting the epistemic condition relies on capacities such as harm recognition, empathy and foresight, and meeting the control condition on, for instance, impulse control, practical reasoning and the motivational strength of moral reasons (Talbert 2024). Importantly, such faculties are generally grounded in more stable psychological dispositions. An aggressive disposition, for example, could cause more rapid anger arousal and increased threat perception, which in turn makes failures in faculties such as impulse control and empathic concern more likely to occur (Cruz et al. 2020). Therefore, a neurointervention used to prevent an offender with a history of aggression from reoffending would likely change not only her aggressive disposition but also faculties such as impulse control and empathy. Seeing that culpability generally tracks such psychological faculties, an intervention that modifies them might transform the substrate on which blame is predicated. Accordingly, the moral basis for holding the offender culpable might become especially questionable if a neurointervention reduces psychological connectedness by changing exactly those psychological dispositions and traits, and associated faculties, that ground culpability. So, in that sense, it seems that diminishing only a small portion of an offender’s overall psychological connections may nonetheless translate to a significant reduction in culpability for the crime that justified the punishment.

## Culpability and the Limits of Justified Punishment

Culpability is, in many penal systems, relevant to justifying punishment. Justifications for punishment differ among countries and cultures, and most Western countries invoke multiple justifications for punishment—such as retribution, rehabilitation and deterrence—rather than relying exclusively on one penal theory (Tonry 2022). As mentioned before, if neurointerventions were to be used in the criminal justice system, it is likely that they would serve rehabilitative aims; they would be used to psychologically ‘reform’ the offender to reduce the likelihood that she will reoffend.^9^ This plausibly also implies that neurointerventions would be used in combination with other punishments serving other punishment goals besides rehabilitation, with the most commonly imposed punishment among Western countries being incarceration (Tonry 2022). Incarceration can satisfy retributivist aims—giving offenders the punishment they ‘deserve’—but also other penal aims such as deterrence or prevention of future harm.

Regardless of what the exact punishment aims are, most penal theories maintain a so-called ‘negative retributivist constraint’ for justified punishment, which holds that punishment should not exceed what an offender deserves, where desert is a function of the severity of the wrongdoing (often tracked by harm caused and wrongfulness of conduct) and the offender’s culpability (Braithwaite and Pettit 1992; Duff 2000; Walen 2021).^10^ This entails that even when punishment is used to achieve other penal goals than retributivist ones, this constraint sets an upper bound, where no individual should suffer more than is proportionate to the severity of the wrongdoing and the individual’s culpability for it. The extent of culpability thus plays an important role in setting a proportionate—and morally justifiable—punishment.^11^ Within most Western criminal justice systems, this means that the length of the prison sentence that would be imposed alongside a rehabilitative neurointervention would be determined, or rather limited, by the extent of culpability.^12^ When culpability diminishes this constraint becomes stricter, where the maximum amount of punishment that remains permissible reduces accordingly.^13^

## Neurointerventions, Psychological Connectedness and Justifying (further) Punishment

If punishment should be proportionate to the extent to which individuals are morally culpable for their crimes, and if a neurointervention effectively reduces psychological connections and culpability, it seems that the extent of punishment that can justifiably be imposed also diminishes.

To illustrate, suppose there is an offender O who has committed multiple violent crimes. For his crimes, he receives a prison sentence of 10 years and is offered a neurointervention for rehabilitative purposes, which he accepts. He undergoes the neurointervention and subsequently serves the rest of his sentence. Now suppose the neurointervention significantly weakens the psychological connections between the offender pre-neurointervention at T^1^, let us call him O^1^, and the offender post-neurointervention at T^2^, or O^2^. The weakened psychological connections might result in a reduction of O^2^’s culpability for the crime that O^1^ committed. However, the culpability level of O^1^ at T^1^ determined the extent of the punishment that seemed justified. If O^2^ post-neurointervention now has to serve the 10-year prison sentence, this seems morally difficult to justify due to the punishment being disproportionate to O^2^’s level of culpability for the punishment-justifying crime that O^1^ committed at T^1^. As established earlier, if neurointerventions successfully diminish or eradicate essential features of that culpability—psychological traits and dispositions associated with past crimes such as violent tendencies—then the total punishment that would be justified is correspondingly less, and it therefore seems morally unjust to require offender O^2^ to serve out the (full) pre-determined sentence based on offender O^1^’s culpability in light of the negative retributivist constraint.^14^

Similar worries regarding justified punishment could be expressed for other—more commonly occurring—cases of reduced culpability. For instance, a significant period of time might reduce psychological connections between a past and a future self, potentially making the future self less culpable for the past self’s crime and therefore less deserving of punishment (Douglas 2019). Indeed, some have questioned the moral grounds for lengthy prison sentences based on the idea that psychological connections and thus culpability weaken over time (Diamantis 2019). In contrast to such ‘natural’ time-driven reductions in psychological connectedness, however, neurointerventions are likely to intentionally target those psychological connections that underpin culpability, since they would alter the psychological traits and dispositions linked to the criminal offense for which one might be blameworthy, as well as the associated psychological faculties relevant to assessing blameworthiness.

Still, other rehabilitative measures such as cognitive or behavioral therapy might also target the psychological traits and faculties that ground culpability, so any remaining punishment after such therapies may likewise be difficult to justify once those traits are modified or eliminated and culpability is reduced. A relevant difference, however, between rehabilitative measures such as cognitive or behavioral therapy and neurointerventions is that therapy tends to induce gradual, cumulative changes over a span of months or years, whereas a neurointervention could bring about significant identity-altering effects abruptly.^15^ One could argue that this temporal disparity does not by itself affect the justification for continued punishment if culpability is reduced, as regardless of whether the change takes place slowly or suddenly, once the offender’s psychological traits or dispositions related to their wrongdoing are significantly altered, the justification for further punishment seemingly weakens. Still, a sudden transformation seems more likely to eliminate a greater degree of overlap in connections, as gradual therapeutic interventions would plausibly leave a ‘thicker’ chain of overlapping psychological connections in place for longer. The abrupt and more extensive losses of connections that neurointerventions might cause arguably pose a greater challenge to preserving culpability.^16^

So, while ‘mere’ reduction of psychological connectedness might already diminish culpability by causing the post-neurointervention offender to be, at least partly, a different person than the pre-neurointervention offender, by targeting exactly those psychological connections that originally grounded culpability for past offenses, neurointerventions also risk cutting the most crucial threads of connectedness for culpability. When punishment set to the pre-neurointervention offender’s culpability level is imposed on the post-neurointervention offender, this punishment may be disproportionate in relation to culpability and therefore no longer morally justifiable.

### Objections

Some may object that a relevant difference between time-induced or therapy-based rehabilitation and neurointervention-based rehabilitation is that the former involve authentic, self-driven psychological changes that result in genuine and sustained moral reform, while the latter might yield immediate results that appear promising at first glance but raise questions about enduring efficacy and moral ownership of the psychological changes (Focquaert and Schermer 2015; Sparrow 2014). The justification for reducing punishment might be more compelling in cases of authentic and genuine moral reform, and thus the ‘engineered’ psychological changes induced by neurointerventions might not provide sufficient moral grounds for culpability reduction because they are not driven by moral reflection, and therefore should not warrant reduced punishment.

First, it is not clear that neurointervention-induced changes cannot develop into stable, morally ‘owned’ traits over time (Levy 2011). For instance, individuals may have tried psychotherapy in vain to modify certain character traits and therefore strongly desire neurointervention-induced psychological changes, which might in that sense be very much ‘owned.’ Hence, psychological changes abruptly caused by neurointerventions need not necessarily fail to reduce culpability based on them being ‘engineered’ or ‘inauthentic.’ Second, if we accept that, as argued earlier, a reduction in psychological connectedness might by itself already weaken culpability due to an offender becoming less connected to their earlier (crime-committing) self—regardless of which connections are changed and whether these involve psychological traits that are crime-related or culpability-defining—the objection that the neurointervention-induced psychological change might not provide sufficient moral grounds for reducing culpability does not seem to hold, as the moral basis for sustaining blame erodes regardless of how the change occurred.

Another objection might come from those who believe that culpability is ‘fixed’ from the moment of wrongdoing (Moore 2010). In that case, any changes in the psychological connections that ground culpability, whether natural or externally induced, would not negate culpability and therefore might not justify a reduction of punishment. However, even if one believes culpability is fixed at the moment of wrongdoing—and the targeting of culpability-grounding connections by neurointerventions does not necessarily reduce it—the mere weakening of psychological connectedness still raises worries about unjust punishment. That is, punishment should be directed at the same offender who ‘earned’ the desert, and when a present offender’s psychological connectedness to the culpable offender weakens due to a neurointervention, a growing share of the punishment post-neurointervention risks being imposed on someone only weakly connected to the offender who bore the initial fixed culpability that grounded desert—meaning that the punishment is (at least partially) misapplied to the wrong person. This mismatch gives a reason to reduce any further punishment even if one maintains that culpability was fixed at the time of the offense.

Lastly, some may object that the chances of neurointerventions seriously undermining psychological connectedness are relatively low, and that neurointerventions disrupting an offender’s psychological links so severely that continued punishment becomes unjust is therefore unlikely. While there are studies that suggest that neurotechnologies can produce significant and abrupt psychological effects (Lipsman et al. 2009; Schüpbach et al. 2006; Thomson et al. 2020), such outcomes are not typical across neurotechnology use (Gilbert et al. 2021). Indeed, not all neurointerventions will have similar and/or serious effects on psychological connectedness, and the worries regarding culpability reductions and justified punishment might therefore not be common or shared among all cases of neurointervention use in criminal justice. For instance, some neurointerventions could have no, or only very modest, effects on psychological connections, or might induce effects as gradual as often seen in cognitive or behavioral therapy (as this has been shown to occur with some neurointerventions (Hescham et al. 2020). Still, the fact that there are cases of sudden psychological connectedness-undermining effects of neurointerventions discussed in the literature seemingly warrants the current evaluation of the implications for justified punishment in light of the possibility that neurointerventions are implemented in criminal justice, as most would consider it highly problematic if even a very small number of innocents (or less deserving) were punished. Moreover, even modest reductions in psychological connectedness can have implications for culpability, and any punishment that exceeds what is proportionate to culpability should raise moral concern. Finally, and perhaps most importantly, because neurointerventions used in criminal justice would modify exactly those psychological traits and dispositions relevant to culpability, it seems that even if a reduction of psychological connectedness on the whole might be modest, this could still translate to a substantial reduction in culpability.

### Alternative Punishment Justifications

The above analysis suggests that further punishment after neurointervention use might, to a certain extent, become morally impermissible if the post-neurointervention offender bears weak(er) psychological connections to the pre-neurointervention offender whose culpability determined the punishment severity—essentially implying that, at least in part, the wrong person might be punished. This conclusion is predicated on the notion that just punishment is punishment that is proportionate to the extent of culpability—i.e., the negative retributivist constraint. While, as mentioned, it is in practice also common for systems with different penal justifications than retributivism to implement the negative retributivist constraint, one could argue that without a notion of just desert, further punishment for offenders after neurointervention-caused reduced culpability may still be justified for other reasons. We might therefore briefly consider what potential effects of neurointerventions on offenders’ psychological connectedness and culpability imply for justified punishment under alternative punishment justifications that do not hinge on retributivist notions.

If the sole justification for punishment were preventing future harm and protecting society, the idea of punishing an offender proportionate to her ‘deserving’ based on her culpability loses force. What is relevant for imposing justified punishment is only the extent to which it prevents future harm, and culpability might then only be relevant for determining *who* should be punished (to prevent the innocent from being punished), not to what extent. Therefore, if neurointerventions would diminish culpability in offenders, this would not necessarily make any further punishment unjust—at least not on the grounds of diminished culpability. Reduced punishment might be warranted on the grounds that an offender is fully rehabilitated after the neurointervention and no longer poses a risk to others (meaning that the goal of punishment to preventing future harm would be achieved). On the other hand, further punishment might still be justified if an offender would still be deemed a danger to society even after a neurointervention weakens psychological connectedness—regardless of whether she is still considered equally culpable for the crime.

If the sole justification for punishment were its deterrent effect for the individual and the general public, culpability, while not directly relevant for determining the extent of punishment one deserves, might still matter in practice, as the perceived fairness of legal threats by the public is often dependent on the level of blameworthiness (Apel and Nagin 2011). Accordingly, if neurointerventions would reduce culpability in criminal offenders it could potentially undercut the rationale for further punishment, as general deterrence might be weakened if the public regards the harsh treatment of a now-less-culpable offender as unfair. Individual deterrence is less dependent on culpability, as while this might lose traction if an offender is now less likely to reoffended and further punishment likely adds almost no additional deterrent effect, it could still be justified to continue punishment if offenders remain at risk of reoffending—irrespective of culpability. A reduction in offenders’ culpability due to neurointerventions may thus also impact the justification for continued punishment under deterrence-based penal theories in terms of general deterrence, although the link between culpability and justified punishment is less explicit than in retributivist frameworks.

So, neurointervention-caused diminished culpability affects justifications for further punishment to a lesser degree under non-retributivist penal justifications than under retributivist ones; continued punishment post-neurointervention therefore seems less morally permissible only if a culpability-based upper limit to justified punishment is adopted, and could potentially be justified on purely deterrent or preventive accounts. (Note that there might be reasons besides diminished culpability why reduced psychological connectedness might call for less punishment on such accounts—for instance, the prospect of psychological disconnection might undermine individual deterrent effects by making people care less about their post-neurointervention self.)

### Practical Considerations

While the issue of unjust punishment due to neurointervention-induced reductions in culpability may thus be less pressing in purely deterrent or preventive frameworks, most contemporary penal systems—not only purely retributivist ones—maintain a culpability-based upper limit on justified punishment, and the problem thus remains practically significant. If neurointerventions rapidly lower culpability, there is a risk of breaching that limit and unjustly inflicting punishment unless the severity is adjusted accordingly.

At this point, some may contend that many penal systems already have procedures in place to adjust the severity of punishment based on psychological or medical factors, for instance via parole, compassionate release, sentence-modification hearings or executive clemency (Padfield et al. 2010; Wylie et al. 2018). Therefore, if neurointerventions would reduce psychological connectedness and thereby culpability to a significant extent, punishment severity would be adjusted and the worry about disproportionate and unjust punishment might thus be overstated. However, these are principally risk-management procedures and tools, not ‘culpability-calibrating’ ones. Parole and related procedures ask whether continued imprisonment is necessary for public safety—they do not assess whether the remaining punishment would exceed what is proportionate to the offender’s current culpability. As a result, they may leave the sentence intact whenever risk is thought to persist—even if culpability has diminished—while sometimes reducing sentences for low-risk individuals whose culpability has not changed. Under the negative-retributivist cap, by contrast, a reduction in culpability seems to impose a moral requirement to reduce any portion of punishment that would otherwise be disproportionate, regardless of residual risk.

Even if these procedures would also take into account culpability, they are designed to deal with ‘ordinary’ psychological and medical changes, and perhaps not the kind of changes that neurointerventions may cause. Consider for instance parole, which is commonly implemented in Western jurisdictions to conditionally release offenders once they have demonstrated sufficient rehabilitation or a low risk of reoffending (Petersilia 2003). Typically, an offender takes part in long-term programs such as counseling, behavioral therapy and educational courses, after which parole boards review the extent to which they believe the offender ready to reintegrate. When offenders are deemed sufficiently ‘reformed’ and no longer a danger to society, they might be allowed to leave prison before serving the complete sentence, reducing their total punishment to a (usually) modest degree (Petersilia 2003). Now, neurointerventions could achieve a level of sufficient ‘reform’ in a short time span very early in the sentence and thereby call into question the fairness of continuing punishment. Parole boards, however, might not be attuned to deal with such abrupt psychological changes, as they typically deal with slow and gradual changes that can be tracked over a longer period of time as observed with traditional rehabilitative measures. Sudden changes induced by neurointerventions might run the risk of, for instance, being viewed as artificial and not a sign of true moral reform by parole boards. Existing evaluation tools might thus not be equipped to assess the specific kind of psychological changes and culpability erosion that neurointerventions might produce, and accordingly, might not be capable of preventing the imposition of unjust punishment on now-less-culpable offenders.

A seemingly simple way to avert the problem of neurointerventions potentially resulting in disproportionate punishment would be to suggest that neurointerventions as rehabilitative measures should be administered *after* offenders have served their full prison sentence, as this would prevent any punishment from being disproportionate to the level of culpability. While this seemingly provides a solution that circumvents the problem, it faces several practical objections with respect to rehabilitative goals. While in a purely retributivist framework it might indeed be defensible to first have offenders serve the predetermined prison sentence and administer neurointerventions after desert has been satisfied, it is likely that those penal systems that would employ neurointerventions would be ones that have rehabilitation as one of the primary aims.^17^ If (one of) the goal(s) is indeed to rehabilitate offenders to facilitate successful reintegration into society, then postponing a potentially rehabilitative and ‘reforming’ measure until after punishment has been completed might unnecessarily delay or obstruct this goal. By postponing rehabilitative neurointerventions, one might risk forfeiting both the immediate and long-term benefits that could come from early rehabilitation. For instance, if administered early in the sentence, neurointerventions could help reduce violence within prison environments. Moreover, there are also considerations regarding rehabilitative desires of offenders. If an offender seeks a neurointervention before or during incarceration, for instance to address their own unwanted violent impulses or psychological problems they experience as undesirable and burdensome, it seems problematic for the state to deny them access (and might even infringe their right to neurorehabilitation if they have one (Dore-Horgan 2023). Thus, even if postponing neurointerventions to after prison sentences have been fully served seemingly avoids the problem of unjustly punishing the less culpable, this is only compelling on purely retributivist frameworks and would arguably obstruct rehabilitative aims that penal systems using neurointerventions presumably would pursue.^18^

## Conclusion

By altering the psychological traits and dispositions that underlie culpability, neurointerventions may reduce the extent to which the post-intervention offender remains culpable for crimes committed by the pre-intervention offender. Since most penal systems recognize a culpability-based limit on justified punishment, any continuation of punishment set to pre-intervention culpability after psychological connectedness has been reduced by a neurointervention may be disproportionate and therefore unjustly imposed on offenders. If neurointerventions are administered for rehabilitative purposes, and if they weaken psychological connectedness and culpability such that further punishment becomes morally impermissible, revision of the original sentence may be warranted.

## Data Availability

Not applicable.

## Footnotes

1 By ‘neurointerventions’ I mean interventions that directly modify neural activity via biophysical or biochemical means (e.g., DBS, tDCS, psychopharmacology). Here, ‘direct’ here refers to a relatively short mechanistic pathway from intervention to neural change, with minimal or no reliance on the subject’s sensory or cognitive engagement. This direct class of neurointerventions offer the clearest test cases for the paper’s arguments about targeted changes in culpability-relevant psychological states and traits, and I thus for the moment set aside ‘indirect’ interventions, such as psychotherapy or behavioral programs, which bring about neural changes through longer, sensory or psychologically-mediated, pathways. Note, however, that directness can be a matter of degree, and some claims may also extend to less direct interventions (with appropriate caveats).

2 Offering implies that an offender must consent to the neurointervention. There are, however, some who doubt whether consent to a neurointervention given by a criminal offender within a criminal justice context can ever reflect truly voluntary—and thus valid—consent due to the coercive background conditions of the context (Focquaert 2014). Moreover, even if consent to neurointervention offers is not automatically undermined by the coercive context, some argue that such offers may still be morally problematic for other reasons—for instance, if the offer is inappropriate in a way that undermines the state’s standing and thus normative consent (Bomann-Larsen 2013) or if the offer involves manipulative pressure (Holmen and Dore-Horgan 2025).

3 While the moral worries arising from ‘identity changes’ might vary for different forms of identity—e.g., numerical and narrative identity—many of the concerns may also overlap, as the leading accounts of both forms of identity typically presuppose some common core of ongoing psychological connectedness—e.g., overlapping memories, dispositions and values—that can support self-reference and persistence over time (Olson 2022; Parfit 1984; Schechtman 2018). When such connections are weakened, similar worries might arise about for instance responsibility for actions or ownership of change, even if narrative accounts emphasize self-interpretation and qualitative aspects while numerical accounts focus on temporal persistence. In the remainder of the article, the focus will be on identity as psychological connectedness over time, which, while commonly understood as an account of numerical identity, is also often presupposed by narrative identity accounts (Schechtman 2018).

4 This would constitute a disruption in psychological *continuity*. Parfit distinguishes psychological connectedness—the direct relations between adjacent temporal stages, such as memories, intentions or beliefs—from psychological continuity, which is an overlapping chain of enough of such connections over time. Connectedness is a scalar notion, where one can have more or fewer direct links between T^1^ and T^2^, while continuity is dichotomous and presents when enough connectedness obtains at each link in the chain and is lacking when the overlap falls below that threshold. Because the arguments in this article require tracking gradations in how much an offender remains the same person—and thus how far culpability persists—I focus on connectedness rather than continuity, consistent with Parfit’s own framework where he analyses continuity in terms of varying degrees of connectedness (Parfit 1984).

5 The claim that moral responsibility presupposes psychological continuity has been disputed by some (e.g., Glannon 1998) on the basis of it having counterintuitive implications such as that lapses of memory would erase desert. Still, not only Parfit but also other theorists often explain diachronic responsibility as depending on some form of psychological connectedness. For instance, Shoemaker’s ‘qualities of will’ account argues that blame is in the agent’s dispositions of regard towards others, and for this stance to hold over time, the later self must be able to endorse the earlier will—which presupposes continuing recognizable traits, attitudes and memories (Shoemaker 2013). Likewise, McKenna’s conversational theory conceives of blame as a form of moral dialogue, and its coherence depends on the person being blamed being stable enough to respond meaningfully to earlier statements (McKenna 2019). Also narrative approaches to identity, such as Schechtman’s (2018), hold that blameworthiness is weakened if an action cannot be integrated into the narrative and the ‘narrative threat’ over time is reduced.

6 Note that in what follows I make no claim that offenders literally become different persons post-intervention. The argument needs only the weaker premise that when connectedness falls below a certain degree, the prudential and moral reasons normally grounded in psychological connectedness—most notably culpability for past wrongdoing—lose strength, even though numerical identity might remain intact. Hence, culpability can diminish without full-blown ‘death’ of the prior self.

7 Sudden and serious effects on identity after neurointervention use might be more rare than the extent of literature on the topic seems to suggest (Pugh 2020). For the present analysis, however, even moderate and gradual effects on identity are relevant, especially if neurointerventions modify traits tightly coupled to culpability such as impulse control or empathy.

8 Some—mostly retributivist-minded—scholars argue that blameworthiness is fixed at the moment of wrongdoing (Moore 2010), and therefore may doubt whether culpability diminishes when psychological connections diminish. This view as an objection is discussed later in the article.

9 Note that there are different definitions of rehabilitation, for a taxonomy see Forsberg and Douglas (2022).

10 While a handful of abolitionist or purely consequentialist proposals reject such a desert ceiling, they remain minority views in practice.

11 There is also positive retributivism and limiting retributivism, the former of which holds that criminal offenders should be punished precisely according to what they deserve (and not more or less), and the latter maintains that for each crime there is a range of proportionate punishments and all punishments within these limits are considered deserved (Walen 2021). Although such positions fit comfortably with strict retributivism, the negative retributivist constraint is more widely accepted even in systems that lean toward non-retributive aims (which are also most likely to employ rehabilitation-oriented neurointerventions).

12 In what follows, I bracket variation in offense severity by assuming a fixed level of offense severity and focus only how changes in culpability may affect the upper bound of justified punishment.

13 A similar concept that has been discussed is the proportionality constraint in relation to an offenders liability, where liability to punishment is understood to be bounded by the same principle of proportionality that the negative retributivist constraint emphasizes: where liability justifies the punishing of the offender, any punishment disproportionate to the offender’s liability—which is generally tied to an offender’s culpability—would be unjust (McMahan 2020).

14 While the focus here is on the negative retributivist constraint, retributivists that endorse the positive claim that there is a duty to realize desert might worry that a ‘retribution gap’ arises once psychological connectedness is weakened, where there is a surplus of desert and no appropriate subject to receive it. Note, however, that this concern only arises for positive retributivists who believe desert is fixed from the moment of wrongdoing and culpability reduction after neurotechnology would thus imply there is no longer an appropriate addressee to receive the desert set at the time of wrongdoing. By contrast, for positive retributivists who allow desert to track current culpability, further punishment would become similarly unjustifiable due to a reduction in culpability implying a proportionate reduction in just desert. I further bracket the (less widely endorsed) positive claim and consider only whether continued punishment would exceed the proportionality cap.

15 Note that it need not always be the case that psychological changes occur gradual along the time span of the cognitive/behavioral therapy, as changes might also occur suddenly (Kelly et al. 2009; Norton et al. 2010). Still, even seemingly abrupt improvements in cognitive or behavioral therapy might emerge only after several hours or weeks of therapy and repeated practice, whereas neurointerventions could shift psychological states within seconds or minutes after stimulation onset. Thus, as a broad rule of thumb it seems plausible to consider therapy to have predominantly gradual effects and neurointerventions as characteristically abrupt in their onset of psychological effects.

16 That the effects of neurointerventions on psychological connectedness lead to greater moral concerns also seems to be corroborated by the fact that ethical concerns regarding so-called ‘identity changes’ following neurointervention use are relatively prominent in the medical and neuroethical literature (e.g., Gilbert et al. 2023; Klaming and Haselager 2013; Lipsman et al. 2009; Lipsman and Glannon 2013; Pugh 2020; Schüpbach et al. 2006), while rarely discussed in the context of cognitive or behavioral therapy (Brewin 2023).

17 Unless neurointerventions would be implemented to inflict harm to in some way give an offender the hard treatment they ‘deserve,’ which, while entertained by some (Ryberg 2025), would in practice be an unlikely use of neurointerventions.

18 As a practical counterexample one may refer to chemical castration, which can be considered a form of neurointervention, and is often offered as a condition of parole (Forsberg 2021a) and thus given *after* desert has been satisfied by (at least most of) the prison sentence. However, this might be a poor analogy for other neurointerventions, as chemical castration is risk-focused rather than rehabilitative; it suppresses testosterone-driven libido and does not target psychological traits or dispositions, and therefore offers little behavioral or moral benefit (especially within prisons). The rationale for implementing it near the point of release is because it is when sexual opportunity returns.
